# *Streptobacillus moniliformis* and IgM and IgG Immune Response in Patient with Endocarditis^1^

**DOI:** 10.3201/eid3003.230917

**Published:** 2024-03

**Authors:** Philipp Mathé, Katja Schmidt, Viktoria Schindler, Ahmad Fawzy, Tilman Schultze, Reinhard E. Voll, David Pauli, Milena Popova, Franziska Schauer, Tobias Eisenberg

**Affiliations:** University Medical Center Freiburg, Freiburg, Germany (P. Mathé, V. Schindler, R.E. Voll, D. Pauli, M. Popova, F. Schauer);; German Cancer Research Center, Heidelberg, Germany (K. Schmidt);; Cairo University, Giza, Egypt (A. Fawzy);; Hessian State Laboratory, Giessen, Germany (A. Fawzy, T. Schultze, T. Eisenberg)

**Keywords:** Rat-Bite Fever, *Streptobacillus moniliformis*, endocarditis, serology, zoonoses, Janeway lesions, rodent, bacteria, One Health, Germany

## Abstract

We describe a case of endocarditis caused by *Streptobacillus moniliformis* bacteria, a known cause of rat-bite fever, in a 32-year-old woman with pet rats in Germany. The patient had a strong serologic response, with high IgM and IgG titers. Serologic analysis is a promising tool to identify *S. moniliformis* bacterial infection.

Rat-bite fever (RBF) is a rare disease that typically manifests with fever, rash, and arthritis (*1*). Possible complications are abscess formation, endocarditis, and death if left untreated (*1*,*2*). *Streptobacillus moniliformis* bacteria is the main causative pathogen of RBF (*3*). Norway rats (*Rattus norvegicus*) are the natural host and usually carry *S. moniliformis* bacteria asymptomatically in their nasopharynx (*3*,*4*). Transmission occurs typically by rat bite or scratch but also by nontraumatic indirect contact.

We describe a case of a 32-year-old woman who came to an emergency department in Germany in May 2022 with fever, fatigue, and migrating arthralgia in the large and small joints of all 4 extremities, without signs of joint swelling or rash. She had a short history of diarrhea, and her first set of blood cultures were negative. She was initially diagnosed with reactive arthritis and transferred to the rheumatology department. We initiated treatment with 20 mg prednisolone and etoricoxib. The patient had initial relief of symptoms and was discharged after 6 days in the hospital. A small papule on her right foot appeared immediately after discharge. A few days later, she went to the dermatology department with a fever and red, nonitching papules on hands, legs, and feet (Figure 1). We examined the papules, finding them comparable to Janeway lesions, and took a biopsy from the right hand. We collected a second blood culture that was positive within 18 hours with growth of a gram-negative bacilli. We identified *S. moniliformis* bacteria by using matrix-assisted laser desorption/ionization time-of-flight mass spectrometry. 

**Figure 1:**
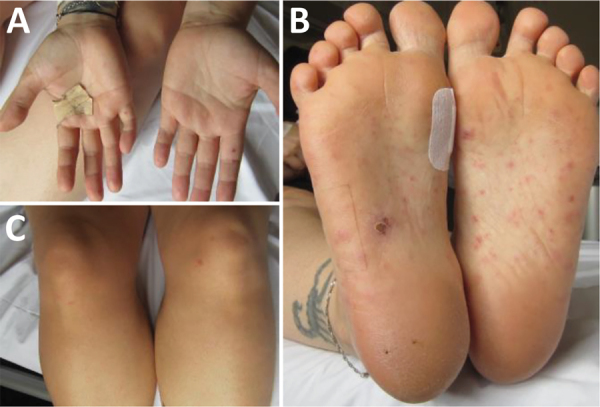
Rat bite fever lesions on 32-year-old female patient, Germany, 2022. At the time of patient’s readmission, reddish papules appeared on the palms of the hands (A), soles of the feet (B), and legs (C).

The patient was readmitted. In an extended history, she reported having 3 Norway rats as pets. Our further investigation revealed an 11-mm size vegetation on the right coronary cusp of the aortic valve; we observed no signs of insufficiency during echocardiography. The patient was diagnosed with RBF and probable aortic valve endocarditis because of meeting 1 major criterion (positive echocardiography) and 2–3 minor criteria (fever, positive blood culture, and suspected Janeway lesions) of the modified Duke criteria (*5*).

After we identified the causative pathogen, we began an intravenous therapy with penicillin G (4 × 5 million IU) for 14 days. Because endocarditis was discovered late in the diagnostic process and no further complications arose, we continued monotherapy under frequent clinical and echocardiographic controls. After 14 days, we changed the therapy to oral amoxicillin (4 × 1 g) for another 4 weeks. Two weeks after the start of oral therapy, we no longer detected the aortic vegetation. Two weeks after therapy concluded, the patient reported well-being and no persistent symptoms.

We used a phylogenetic approach to group the microorganism from this study to closely related taxa (Appendix). The resulting tree confirmed the taxonomic position of the isolate from this study as a member of *S. moniliformis* bacteria.

In addition to microbiologic work-up, we analyzed serum samples from different time points for *S. moniliformis* bacteria–specific antibodies by using *Streptobacillus* multiplex serologic analysis. We found high IgM and IgG antibody levels in the patient’s serum 9 days after symptom onset. IgM levels of subsequent measurements decreased, and IgG levels initially increased before declining approximately 3 weeks after the onset of symptoms (Figure 2).

**Figure 2:**
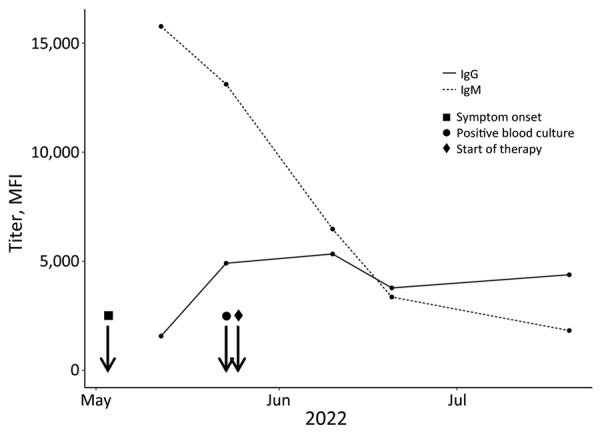
Antibody response to *Streptobacillus moniliformis* infection over time on 32-year-old female patient, Germany, 2022. The graph displays the dynamics of IgM (serum dilution 1:100) and IgG (serum dilution 1:250) levels in MFI values analyzed by *Streptobacillus* multiplex serologic tests (y-axis) and plotted against the time point of infection (x-axis). MFI, median fluorescence intensity.

Several aspects hamper the diagnosis of RBF, including unawareness of the disease among most clinicians, lack of reliable diagnostics, fastidious growth of the microorganism, susceptibility to most antibiotics used for empiric therapy (*3*), and unnoticed animal contact (*6*). Therefore, the incidence of RBF is unknown and difficult to estimate, especially because RBF is a nonnotifiable disease worldwide. Most of the published case reports do not properly identify the causative organism because they rely solely on 16S rRNA gene sequencing, which is insufficient for an accurate identification at species level (*6*).

In cases where direct detection methods, such as pathogen isolation or molecular testing, are not successful, serologic analysis could be a useful tool for clinical decision-making. High initial IgM and IgG levels of *S. moniliformis* bacteria–specific antibodies were measured in the patient by using *Streptobacillus* multiplex serologic analysis. However, because serologic tests for *S. moniliformis* bacteria are not commercially available nor readily accessible, the prevalence of RBF among humans is unknown. Further serologic studies could help to estimate the occurrence of RBF by shedding light on a largely unknown and underreported disease (*6*). Novel PCR tools could help to reduce the number of undetected infections and enable appropriate treatment.

This case report highlights the benefits of a One Health approach to healthcare in daily practice. Veterinary healthcare provided valuable information for clinicians regarding this rare disease and provided a serologic assay originally developed for the health monitoring of laboratory rodents and adapted for human application. Population-level serologic studies are needed to assess disease prevalence in high-risk groups. This case shows the possibility of species-specific RBF diagnosis in cases where direct diagnostic tools prove to be negative. 

Appendix Additional information for *Streptobacillus moniliformis* and IgM and IgG immune response in patient with endocarditis.
