# Supplementary material for: Streptobacillus moniliformis and IgM and IgG Immune Response in Patient with Endocarditis
Source: Emerg Infect Dis. 2024 Mar;30(3):608–10. doi: 10.3201/eid3003.230917 (PMC10902545; doi:10.3201/eid3003.230917)
Supplement: Appendix — Additional information for Streptobacillus moniliformis and IgM and IgG immune response in patient with endocarditis. [file 23-0917-Techapp-s1.pdf]

Article DOI: https://doi.org/10.3201/eid3003.230917

Isolation of *Streptobacillus moniliformis* and IgM and IgG Immune Response in Patient with Endocarditis

Appendix

Methods used for phenotypic and molecular characterization of the *S. moniliformis* isolate

Phenotypic characterization

Bacterial isolation and physiologic properties

Bacterial isolates were identified using standard microbiological examination procedures. Briefly, venous aerobic and anaerobic blood cultures were incubated at 37°C, showing growth after 22 hours in the aerobic culture and after 4 days in the anaerobic culture. Isolates were subcultured on solid agar media (Columbia agar with 5% sheep blood [SBA; Oxoid, Wesel, Germany]. Agar plates were incubated for up to 48 h at 37°C using aerobic and microaerobic culture conditions. Phenotypic characterization of streptobacilli is known to yield only few weakly positive reactions (*1*), however, standard microbiological procedures included tests for hemolysis on SBA, catalase activity with 3% H_2O_2 on microscopic slides and for the presence of cytochrome oxidase with the BBL DrySlide® oxidase system (Becton–Dickinson, Heidelberg, Germany). Urease, hydrogen sulfide, indole, motility and oxidative and fermentative glucose assimilation were tested on Christensen agar, SIM and OF medium in slant agar tubes, respectively (all Merck, Darmstadt, Germany). Microscopic examinations of fixed smears were performed using Gram stain.

Matrix-assisted laser desorption/ionization-time of flight mass spectrometry (MALDI-TOF MS)

For further identification attempts, the isolate was investigated by MALDI-TOF mass spectrometry. Mass spectrometry procedure has recently been described in detail (*2*,*3*). The commercial database used (DB 8,468; BrukerDaltonics) comprised 24 spectra each from 10 *S. moniliformis* strains. Reference spectra from well-characterized, quality-controlled strains of all

other *Streptobacillus*/*Pseudostreptobacillus* species and most other members of the *Leptotrichiaceae* were added to the database from previous studies (*2*,*4*). Identification was carried out with the commercial Bruker database, and with the extended database.

Molecular characterization

Whole genome sequencing and phylogenomic analysis

Whole genome sequencing (WGS) was carried out to get insight into a core genome based phylogeny and compare the patient's isolate with established field and type strain genomes from the same genus. The genome sequence of isolate 221009635 was determined by de-novo assembly with reads from Illumina technology. In brief, DNA was isolated from cells grown for 3 days at 37°C on SBA using a PureLink genomic DNA kit (Thermo Fisher). The library was prepared with DNA prep library preparation kit (Illumina) and sequenced on MiSeq instruments using v3 chemistry and a paired-end sequencing approach of 2×300 bp. Generated sequence data are deposited in the NCBI sequence read archieve (SRA) under the BioProject accession PRJNA946922. The genome assembly and quality assessment were carried out using the Aquamis pipeline (vers. 1.3.7), resulting in 179 contigs with 90x average coverage. Whole genome annotation was performed using prokka (vers. 1.14.6) (*5*).

A core genome phylogeny of strain 221009635 and 39 genomes of the genera *Streptobacillus/Pseudostreptobacillus* was calculated in EDGAR 3.0 based on MUSCLE alignment as previously described (*6*). This resulted in one multiple alignment of 481 core genes per genome (19,240 genes in total), with 169,393 aa residues per genome (6,775,720 in total). ANI values were computed as described by Goris et al. (*7*) and as implemented in JSpecies (*8*). The Neighbor-Joining algorithm was used for tree generation.

Species identity between the isolate and 27 other *S. moniliformis* genomes was confirmed by mean average nucleotide identity (ANI) values of >98.9% (data not shown), which is clearly above the >95%–96% proposed boundary for identical species (*7*). Based on an earlier published MLVA scheme for typing *S. moniliformis* (*9*) and by using WGS data, the isolate depicted the same genotype LHL11 like strains NCTC 11941 and Kun 3 (RIVM).

Methods used serologic investigation

Serology

The *S. moniliformis* serologic assay is part of the multiplex serology, which is used for routine health monitoring of laboratory rodent colonies at the DKFZ. The multiplex serology is based on an immunosorbent assay in combination with the fluorescent bead technology from Luminex Corp. (Austin, TX, USA). Membrane proteins of *S. moniliformis* Levaditi et al. type strain (ATCC 14647) were extracted and directly coupled to magnetic beads with an embedded fluorescent dye (MagPlex® Microspheres; Luminex Corp.) before incubation with serum (final dilution 1:100 for IgM and 1:250 for IgG analyses). The general set-up and protocol of the *Streptobacillus* multiplex serology is described by Schmidt et al. (manuscript in preparation). The Luminex analyzer BioPlex200 (BioRad Laboratories GmbH, Munich, Germany) was used to distinguish between the bead set and the bound antigen, and to quantify the amount of bound serum antibody by a secondary antibody (1:1000 dilution of biotinylated goat anti-human IgM or goat anti-human IgG, Jackson ImmunoResearch Laboratories, Inc., West Grove, PA, USA) and a fluorescent reporter conjugate (1:500 Streptavidin-R-phycoerythrin). Final antigen-specific median fluorescence intensity (MFI) values were measured of at least 50 beads per bead set and serum sample.

References

1. Eisenberg T, Ewers C, Rau J, Akimkin V, Nicklas W. Approved and novel strategies in diagnostics of rat bite fever and other *Streptobacillus* infections in humans and animals. Virulence. 2016;7:630–48. PubMed https://doi.org/10.1080/21505594.2016.1177694

2. Eisenberg T, Heydel C, Prenger-Berninghoff E, Fawzy A, Kling U, Akimkin V, et al. *Streptobacillus canis* sp. nov. isolated from a dog. Int J Syst Evol Microbiol. 2020;70:2648–56. PubMed https://doi.org/10.1099/ijsem.0.004086

3. Eisenberg T, Glaeser SP, Blom J, Rau J, Kämpfer P. *Streptobacillus*. In: Bergey's manual of systematics of archaea and bacteria. John Wiley & Sons, Ltd; 2018. p. 1–13 [cited 2023 Apr 22]. https://onlinelibrary.wiley.com/doi/abs/10.1002/9781118960608.gbm00774.pub2

4. Rau J, Eisenberg T, Männig A, Wind C, Lasch P, et al. MALDI-UP—an internet platform for the exchange of MALDI-TOF mass spectra. User guide for http://MALDI-UP.ua-bw.de. Aspects of

Food Control and Animal Health 2016:1–17 [cited 2022 Dec 5]. https://ejournal.cvuas.de/docs/cvuas_ejournal_201601.pdf

5. Seemann T. Prokka: rapid prokaryotic genome annotation. Bioinformatics. 2014;30:2068–9. PubMed https://doi.org/10.1093/bioinformatics/btu153

6. Blom J, Kreis J, Spänig S, Juhre T, Bertelli C, Ernst C, et al. EDGAR 2.0: an enhanced software platform for comparative gene content analyses. Nucleic Acids Res. 2016;44(W1):W22-8. PubMed https://doi.org/10.1093/nar/gkw255

7. Goris J, Konstantinidis KT, Klappenbach JA, Coenye T, Vandamme P, Tiedje JM. DNA-DNA hybridization values and their relationship to whole-genome sequence similarities. Int J Syst Evol Microbiol. 2007;57:81–91. PubMed https://doi.org/10.1099/ijs.0.64483-0

8. Richter M, Rosselló-Móra R. Shifting the genomic gold standard for the prokaryotic species definition. Proc Natl Acad Sci U S A. 2009;106:19126–31. PubMed https://doi.org/10.1073/pnas.0906412106

9. Eisenberg T, Fawzy A, Nicklas W, Semmler T, Ewers C. Phylogenetic and comparative genomics of the family *Leptotrichiaceae* and introduction of a novel fingerprinting MLVA for *Streptobacillus moniliformis*. BMC Genomics. 2016;17:864. PubMed https://doi.org/10.1186/s12864-016-3206-0

Appendix Table. Results of antimicrobial sensitivity testing using ETests (Liofilchem®)

Antibiotic agent	MIC (mg/l)
Penicillin G	0.25
Amoxicillin + Clavulanic acid	0.38
Ceftriaxon	0.023
Doxycyclin	≤0.016
Ciprofloxacin	0.19
Levofloxacin	0.19
Gentamicin	1
Rifampin	0.032

No susceptibility breakpoints exist for *Streptobacillus moniliformis*.

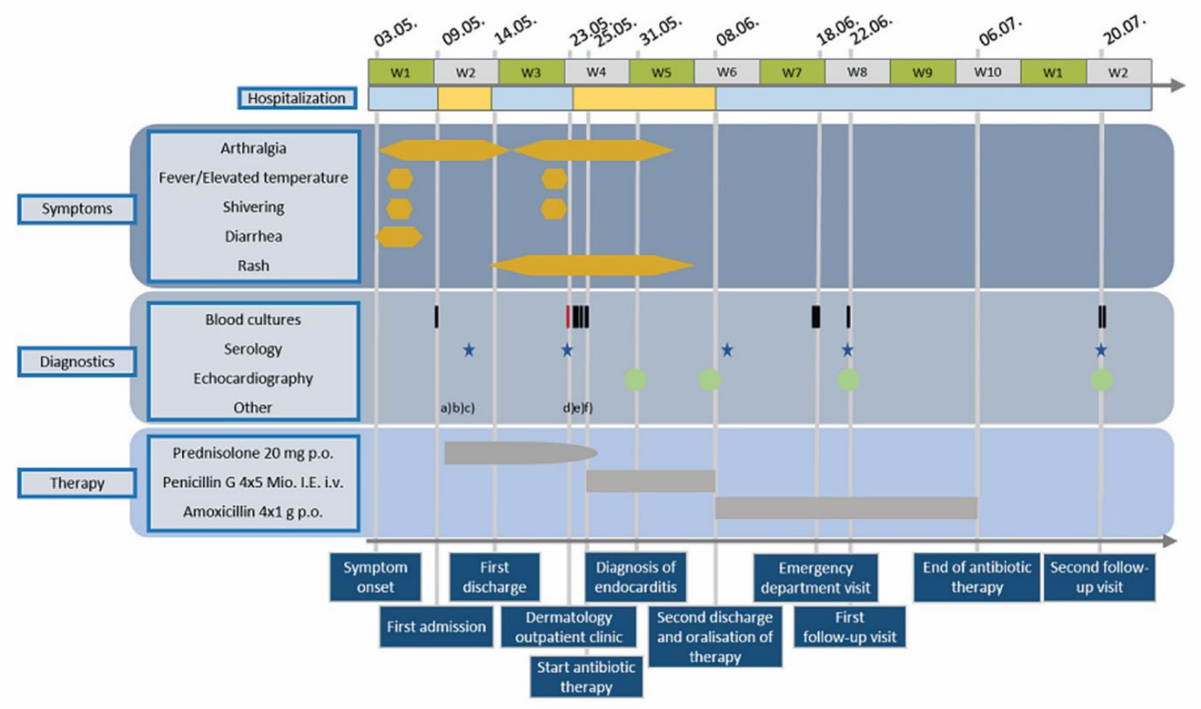

Appendix Figure 1. Time course of the *Streptobacillus moniliformis* infection from this study. The time course of the infection including performed diagnostic tests and results are displayed. The red bar in the diagnostics section represents the time point of the positive blood culture.

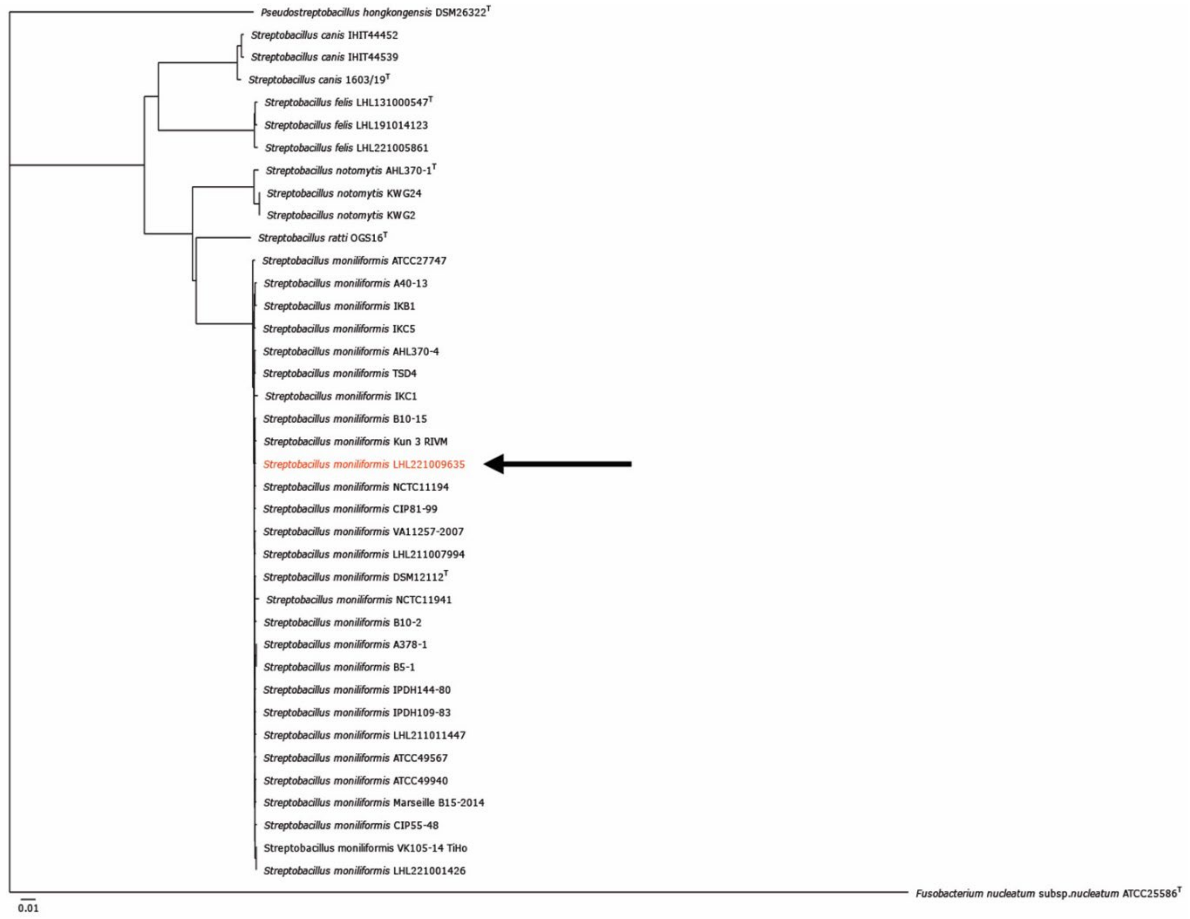

Appendix Figure 2. Core genome phylogenetic tree including the isolated *Streptobacillus moniliformis* strain. The core genome phylogenetic tree based on amino acid sequences depicts *Streptobacillus moniliformis* isolate 221009635 from this study within the genera *Streptobacillus/Pseudostreptobacillus*. Core genes of these genomes were computed in EDGAR 3.0 based on MUSCLE alignments and the Neighbor-Joining algorithm as implemented in the PHYLIP package. *Fusobacterium nucleatum* ssp. *nucleatum* is used as outgroup. Bar, 0.01 amino acid substitutions per site.
